# The Impact of Relationship Satisfaction With Adult Children on Depression Among Older Adults With Abusive Spouses

**DOI:** 10.1093/geroni/igaa057.154

**Published:** 2020-12-16

**Authors:** Sukyung Yoon

**Affiliations:** University of Wyoming, Laramie, Wyoming, United States

## Abstract

Adults (65 and older) comprised about 15% of the 2019 South Korean population (hereafter Korea), but are estimated to be 20% in 2025 and 40% in 2050 (StatisticsKorea, 2019). Good relationships with spouses impact mental health during later life (Santini et al., 2015) but 10.2% of women and 7.6% men 65 and older reported they experienced spousal violence (The Domestic Violence Survey, 2016) Moreover, violent behavior in baby-boomer marriages was significantly higher than their counterparts (Suh, 2015). Previous research investigated how relationships with adult-children impacted older Koreans’ mental health(Kim & Ko, 2013) but few examined the influence on older-adults with abusive spouses. This study investigates depression among older-adults with abusive spouses, and the impact of relationship-satisfaction with their adult-children on depression. This study utilizes the 13th wave of the nationally representative Korea Welfare Panel (2018). The sample consisted of 353 older adults 65 and older with abusive spouses over the past year. The dependent variable was depression, measured using the CES-D-11. The relationship-satisfaction with adult-children was measured on a seven-point Likert scale. Education, health, religion, sex, and age were included, and multiple regression analysis was conducted. The relationship-satisfaction with adult-children and good health status were significantly reduced depression among the population. Health care professionals and practitioners should screen for elder abuse and depression. Additionally, programs are needed to help older - adults develop good relationships with family members are needed.

